# Protocatechualdehyde attenuates oxidative stress in diabetic cataract via GLO1-mediated inhibition of AGE/RAGE glycosylation

**DOI:** 10.3389/fphar.2025.1586173

**Published:** 2025-06-25

**Authors:** Xiao Cheng, Sixuan Zhao, Yucai Chen, Jiawei Wang, Furong Han

**Affiliations:** ^1^ Department of Pharmacy, Beijing Tongren Hospital, Capital Medical University, Beijing, China; ^2^ School of Traditional Chinese Medicine, Beijing University of Chinese Medicine, Beijing, China

**Keywords:** Protocatechualdehyde, Glo1, AGEs/RAGE, oxidative stress, diabetic cataract

## Abstract

**Background:**

Protocatechualdehyde (PCA), a phenolic compound derived from *Salvia miltiorrhiza*, exhibits anti-proliferative and antioxidant properties. However, its molecular mechanisms in reducing oxidative stress in diabetic cataract (DC) remain unclear. This study systematically investigated the role of PCA in modulating glyoxalase-1 (GLO1)-dependent suppression of advanced glycation end product (AGE)-receptor for AGE (RAGE) axis activation and oxidative stress in DC models.

**Methods:**

A galactose-induced DC rat model and high glucose-stimulated human lens epithelial cells (HLECs) were employed. Lens opacity was assessed using slit-lamp microscopy. GLO1, AGE, and RAGE expressions were analyzed through immunohistochemistry (IHC), immunofluorescence (IF), ELISA, and Western blotting. Molecular docking was performed to validate PCA–GLO1 interactions.

**Results:**

PCA administration (25 mg/kg) significantly alleviated lens opacity and epithelial cell disorganization in DC rats (p < 0.01). *In vitro*, PCA (10 μM) restored HLEC viability under hyperglycemic conditions (p < 0.05). Mechanistically, PCA upregulated GLO1 expression while suppressing AGE accumulation and RAGE activation in both models. Molecular docking revealed strong binding affinities between PCA and GLO1 (-CDOCKER energy: 26.41 kcal/mol).

**Conclusion:**

PCA ameliorates DC progression by enhancing the GLO1-mediated detoxification of AGE precursors, thereby inhibiting AGE/RAGE-driven oxidative stress. These findings provide a foundation for PCA as a therapeutic candidate for DC.

## 1 Introduction

Diabetic cataract (DC), characterized by accelerated lens opacification, represents a prevalent ocular complication in diabetic patients, with a five-fold higher incidence than in non-diabetic populations (Stepp and Menko, 2021; Kiziltoprak et al., 2019). Despite surgical intervention being the primary treatment, postoperative complications such as macular edema and secondary glaucoma necessitate the development of pharmacological strategies to delay DC progression (Hilliard et al., 2020). Pathologically, DC is driven by chronic hyperglycemia-induced pathways, including aldose reductase activation, sorbitol accumulation, and oxidative stress amplification via the AGE/RAGE axis (Liu et al., 2020; Armao et al., 2019; Tao et al., 2022). Among these, the irreversible formation of AGEs and subsequent RAGE-mediated NADPH oxidase (Nox)/NF-κB activation constitute a self-perpetuating cycle of oxidative damage, even under glycemic control (Zych et al., 2020; Antonetti et al., 2021).

Glyoxalase-1 (GLO1) is a rate-limiting enzyme in the glyoxalase system that detoxifies methylglyoxal (MGO), a primary precursor of AGEs (Dehnad et al., 2020). The downregulation of GLO1 in hyperglycemic conditions exacerbates AGE accumulation and RAGE signaling (Ruiz et al., 2020; Truong et al., 2019). GLO1 deficiency exacerbates MG-derived glycative stress, impairing redox homeostasis and activating pro-inflammatory pathways such as NF-κB and NLRP3 inflammasomes, which are implicated in lens epithelial cell (LEC) apoptosis and epithelial–mesenchymal transition (EMT) (Hu et al., 2022). Notably, EMT in LECs—a hallmark of posterior capsular opacification (PCO)—is driven by TGF-β/Smad signaling and extracellular matrix (ECM) remodeling, which are processes amplified by AGE–RAGE axis activation (Hu et al., 2022; Liu et al., 2023). For instance, in diabetic cataract models, GLO1 downregulation correlates with increased α-SMA and fibronectin expression, indicative of LEC transdifferentiation into myofibroblasts (Hu et al., 2022). Furthermore, oxidative stress from impaired GLO1 activity compromises the blood–aqueous barrier, exacerbating intraocular inflammation and accelerating PCO progression (Liu et al., 2023). These findings position GLO1 as a dual regulator of glycative and oxidative damage in lens pathology, offering a mechanistic bridge between metabolic dysfunction and structural degeneration. Targeting GLO1 may thus represent a novel strategy of preserving lens transparency and mitigating post-surgical complications.

Protocatechualdehyde (PCA), a bioactive polyphenol from *Salvia miltiorrhiza*, has demonstrated anti-glycation and antioxidant effects in diabetic complications (Pang et al., 2020; Chaudhuri et al., 2018). Recent studies have demonstrated that PCA mitigates oxidative stress by suppressing phagocytic oxidative burst in polymorphonuclear neutrophils and macrophages, thereby reducing reactive oxygen species (ROS)-mediated lens epithelial cell (LEC) apoptosis and crystallin cross-linking (Erukainure et al., 2017). In diabetic models, PCA enhances extracellular matrix (ECM) stability by upregulating cartilage-specific genes (e.g., collagen II and aggrecan) while suppressing dedifferentiation markers like collagen I—a mechanism that may analogously preserve lens transparency by maintaining crystallin integrity (Luo et al., 2015). Furthermore, PCA improves lipid metabolism via peroxisome proliferator-activated receptor alpha (PPARα) activation, reducing lipid peroxidation—a key contributor to lens opacification (Ding et al., 2024). These effects align with its broader role in diabetic complications, where PCA reshapes gut microbiota to reduce systemic inflammation and oxidative damage, which are pathways implicated in diabetic cataract progression (Ding et al., 2024). Collectively, PCA’s ability to modulate AGE/RAGE signaling, enhance antioxidant defenses, and stabilize structural proteins positions it as a novel intervention for diabetic cataract, warranting further exploration in ocular-specific models.

Therefore, based on the above, this study hypothesizes that PCA attenuates oxidative stress by enhancing the GLO1 activity to disrupt the AGE/RAGE axis. This research integrates *in vivo*, *in vitro*, and *in silico* approaches to elucidate the therapeutic potential of PCA in DC.

## 2 Materials and methods

### 2.1 Animal model and drug administration

Male Wistar rats (48 rats, 60 g ± 5 g) were obtained from Beijing HFK Biotechnology Co., Ltd. (Beijing, China) and housed under controlled conditions (23°C ± 2°C, 55% ± 5% humidity, 12-h light/dark cycle) with *ad libitum* access to water and standard chow. After acclimatization for 3 days before the experiment, a DC model was established. All animal care and experimental procedures were approved by the Committee of Ethics of Animal Experimentation of Beijing University of Traditional Chinese Medicine (Approval number: BUCM-1-2024062001-0016).

PCA was prepared as a 2.5-mg/mL solution with normal saline and administered at 25 mg/kg body weight. The *in vivo* dose of PCA (25 mg/kg) was selected based on prior studies that demonstrated their efficacy and safety in comparable experimental models. In rodent studies, 25 mg/kg PCA has been shown to achieve plasma concentrations sufficient to modulate oxidative stress and inflammatory cytokines without adverse effects, aligning with its therapeutic window in metabolic contexts (Semaming et al., 2015; Ji et al., 2021). Galactose for intraperitoneal injection was prepared in sterile water with a 50% concentration solution. The dosage was calculated daily and prepared on a sterile table. The galactose drinking water was prepared as a 5% solution with normal saline and replaced regularly to prevent bacterial growth.

This experiment involved three groups of rats, with 16 rats per group. In the control group, daily intraperitoneal injections of saline were given from day 1. In the model and PCA groups, a 50% galactose solution of 1 mL/100 g body weight was intraperitoneally injected from days 1 to 7. From days 8 to 14, 50% galactose solution was intraperitoneally injected at 1.5 mL/100 g body weight. From days 15 to 26 (experimental endpoint), 50% galactose solution was intraperitoneally injected with 2 mL/100 g body weight. In addition, in the model and PCA groups, 5% galactose water was freely consumed from day 8 onward. Finally, in the PCA group, PCA was given daily by intragastric administration at a dose of 25 mg/kg, in addition to the modeling procedure. At the same time, the control and model groups were given the same volume of normal saline by gavage.

The galactose-induced DC model was selected for its ability to recapitulate key pathological features of chronic diabetic complications, particularly those mediated by oxidative stress and AGEs. Unlike classical streptozotocin (STZ)-induced hyperglycemia, which rapidly destroys pancreatic β-cells to mimic acute hyperglycemia, galactose feeding induces sustained intracellular galactitol accumulation via the polyol pathway (Kador and Kinoshita, 1984). This process mirrors the gradual metabolic dysregulation observed in long-term diabetes, leading to mitochondrial dysfunction, ROS overproduction, and AGE formation—hallmarks of diabetic complications such as neuropathy, retinopathy, and nephropathy (Brownlee, 2001). Furthermore, galactose avoids the off-target cytotoxicity of STZ, which can independently damage non-pancreatic tissues (e.g., kidneys and liver), confounding mechanistic studies (Brownlee, 2001). The galactose model has been extensively validated for studying AGE-driven pathologies and is particularly suited for evaluating interventions that target oxidative stress or glycation pathways. For instance, studies have demonstrated its utility in replicating diabetic ocular lesions and neuronal damage, aligning with our focus on chronic complication mechanisms (Kador and Kinoshita, 1984; Brownlee, 2001).

### 2.2 Body weight

The body weight was recorded during the experimental period to evaluate the effects of PCA on the growth performance of rats with diabetic cataract.

### 2.3 Detection of lens opacity in rats

At the end of the experiment, after atropine-induced mydriasis, the left and right eyes of the rats in each group were photographed using a slit-lamp microscope and were scored. The scoring standard for the severity of lens opacity in rats is divided into five levels. Grade I: the lens is clarified; Grade Ⅱ:vacuoles appear around the lens; Grade Ⅲ: vacuoles disappear, and the lens cortex appears hazy; Grade Ⅳ: the lens cortex is completely cloudy, but the nucleus is transparent; Grade Ⅴ: the nucleus of the lens also appeared cloudy and is gradually mixed with the cortex. The higher the level, the higher the degree of opacity.

### 2.4 Detection of the lens epithelium by HE staining in rats

After the rats were sacrificed, the lenses were fixed in 4% paraformaldehyde overnight, and paraffin sections were prepared. The lens sections were dewaxed with a xylene and ethanol solution of different concentrations, cleaned with phosphate-buffered saline (PBS), stained with hematoxylin solution for 10 min, differentiated in 1% hydrochloric acid for 5 s, rinsed with the eosin dye solution for 5 min after washing with PBS, dehydrated with ethanol solution after staining, made xylene transparent, and finally dried and sealed with neutral resin. The pathological morphology of the lens epithelium was observed under an optical microscope.

### 2.5 Detection of proteins in the lens by immunohistochemistry

After the rats were sacrificed, their lenses were fixed with 10% paraformaldehyde. Then, the samples underwent paraffin embedding, dewaxing, and hydration. Next, H_2_O_2_ was added to block endogenous peroxidase activity, and then antigen repair. Subsequently, GLO1, AGE, and RAGE antibodies were added and incubated overnight at 4°C. After that, a second antibody was added and incubated at room temperature. A 3,3′-diaminobenzidine (DAB) working solution was added for 5–10 min to develop color, and the lenses were re-dyed, dehydrated, made transparent, and sealed. Finally, the images were randomly collected via optical microscope.

### 2.6 Detection of proteins in the lens by immunofluorescence

After the rats were sacrificed, their lenses were fixed with 10% paraformaldehyde, then were paraffin-embedded, sliced, dewaxed, antigen-repaired, sealed with goat serum, and incubated at room temperature for 2 h. After that, GLO1, AGE, and RAGE antibodies were added and incubated at 4°C overnight, and then secondary antibodies were added and incubated at room temperature for 2 h away from light. After staining with 4′,6-diamidino-2-phenylindole (DAPI), the samples were rinsed with PBS, the anti-fluorescence quenching tablets were sealed, and the images were observed and photographed under an inverted fluorescence microscope.

### 2.7 Detection of proteins in the lens by Western blotting

A rat lens was placed in a homogenizer, protein extract was added for homogenization, and a protein sample was prepared. After the protein concentration was detected by a bicinchoninic acid (BCA) kit, the samples were taken for sodium dodecyl sulfate polyacrylamide gel electrophoresis (SDS-PAGE) and transferred to an ice bath. The transferred polyvinylidene fluoride (PVDF) membrane was incubated in the sealing solution at room temperature for 2 h, the primary antibody (including GLO1, AGEs, RAGE antibodies) was added, the secondary antibody was incubated at room temperature for 2 h, and the film was washed and incubated at room temperature for 2 h. Finally, luminescent reagents were added for color development, and images were captured and analyzed using a gel imager. The internal reference protein was β-actin.

### 2.8 Detection of oxidative stress in the lens by ELISA

Rat lens was put into a grinder and mashed with the correct amount of normal saline, centrifuged at 1,000*g for 10 min, and the supernatant was taken. Then, in accordance with the operation steps of the ELISA kit, respectively, detection was made of the lens catalase (CAT), glutathione peroxidase (GPX), super oxide dismutase (SOD), advanced oxidation protein products (AOPP), oxidized glutathione (GSSG), thiobarbituric acid reactive substances (TBARS), and GLO1 expression levels.

### 2.9 Cell culture and treatment

Human lens epithelial cells (ATCC CRL-11421) were cultured in low-concentration glucose DMEM containing 10% fetal bovine serum and placed in a constant temperature incubator containing 5% CO_2_ at 37°C. The logarithmic growth period and a fusion degree 70%–80% of the cells were used in the experiment.

When the cell adhesion growth reached 80%, it was treated with medicine. The original medium was extracted, and 10% fetal bovine serum (FBS) low-glucose medium (100 μL/well) was added to the Control group, 10% FBS high-glucose medium (100 μL/well) was added to the Model group, and 10% FBS high-glucose medium + PCA (10 μM, 100 μL/well) was added to the PCA group. The cells were incubated at 37°C and 5% CO_2_ for 48 h. The *in vitro* concentration (10 μM) was selected based on prior studies that demonstrated their efficacy and safety in comparable experimental models. For *in vitro* experiments, 10 μM PCA has consistently shown dose-dependent inhibition of key signaling pathways (e.g., AGE–RAGE axis) in epithelial cell lines, ensuring biological relevance while avoiding cytotoxicity (Semaming et al., 2015; Wang et al., 2014).

### 2.10 Cell viability assay

The morphological changes of the cells were observed and photographed after the drug treatment was completed. First, a Cell Counting Kit-8 (CCK8) was diluted with medium (CCK8: medium = 1:10). Then, the old culture medium of the cells in each well was extracted, and 100 μL CCK8 diluent was added to each well. Then, the cells were put into the incubator for 2 h. Finally, the OD values of each well were measured at 450 nm by an enzyme-labeled instrument.

### 2.11 Detection of proteins in human lens epithelial cells by immunofluorescence

Immunofluorescence detection was performed after the cell drug treatment. The original medium was extracted and cleaned once with PBS or normal saline for 3 min. We added 100 μL 4% paraformaldehyde into each well and fixed it at room temperature for 20 mi and then cleaned it with PBS or normal saline twice for 3 min each time. We used 0.2% TritonX-100 100°μL/well at room temperature to break the film for 15 min, then 3% BSA, 100 μL per well, closed at 37°C for 30 min. Primary antibodies (AGEs, RAGE, GLO1, and 3-nitrotyrosine (3-NT)) were added and formulated at 1:100, 50°μL/well. It was incubated at 4°C overnight. The primary antibody was recovered and lightly washed with PBS or normal saline three times for 3 min each time. Alexa Fluor 555 coupled secondary antibody was added, prepared at 1:200, 70°uL/well. It was incubated at 37 °C for 2 h in the dark., Hoechst 33342 dye solution of 10°μL/well staining was added to the incubated secondary antibodies prepared at 1:100 10–15 min before photography and then washed with PBS or saline thrice, 3 min each time. Pictures were taken and saved as soon as possible.

### 2.12 Detection of proteins in human lens epithelial cells by Western blotting

Protein samples were prepared by adding the RIPA lysate into the cells. After the protein concentration was detected by the BCA kit, the samples were taken for SDS-PAGE gel electrophoresis and ice bath transfer. The transferred PVDF membrane was incubated in the sealing solution at room temperature for 2 h, the primary antibody (including 3-NT, GLO1, AGE, and RAGE antibodies) was added, the secondary antibody was incubated at room temperature for 2 h, and the film was washed and incubated at room temperature for 2 h. Finally, luminescent reagents were added for color development, and the gel imager took photos and analyzed the protein content. β-actin protein was used as the internal reference protein.

### 2.13 Detection of ROS in human lens epithelial cells

ROS detection was performed after the cell drug treatment. They were first diluted with 10× buffer with normal saline to 1× buffer. Then, the cells were washed with buffer diluent of 100 μL per well for 2 min. After that, the DCFH-DA dye solution was diluted at 1:1,000, 70 μL per well and incubated at 37°C away from light for 45 min. We then discarded the dye solution, added the 1:1,000 diluted Hoechst 3342 dye solution, 100 μL per well, and hid it from light at 37°C for 10 min. Next, they were washed with a buffer diluent of 100 μL per well twice, 3 min each time. Finally, 100 μL buffer diluent was added to each well, and the stained cells were observed under a fluorescence microscope.

### 2.14 Molecular docking

First, a small molecular compound protocatechualdehyde structure was retrieved from PubChem (https://pubchem.ncbi.nlm.nih.gov/). The Protein Data Bank (PDB) database (https://www.rcsb.org/structure/) was then used to obtain GLO1 protein crystal structure. We selected three GLO1 proteins with different structures (PDB ID:2ZA0, PDB ID:3VW9, and PDB ID:7WT0) for molecular docking. We removed excess sequences, water, and ligands from the protein crystal structure. Molecular docking was performed using Discovery Studio 4.5 software. After that, the chemical structure of the small-molecule compound was set as a ligand, and the CHARMM general force field was added. CDOCKER DOCK was performed using Discovery Studio 4.5 software, 2D and 3D visualizations were saved, and the binding energy between small molecules and GLO1 proteins was calculated.

### 2.15 Molecular dynamics simulation

The PDB database was used to find the target Protein GLO1 3D structure, which was downloaded as in PDB format. Then, molecular docking was performed using the obtained protein crystal structure. We removed excess sequences, water, and ligands from the protein crystal structure. Molecular docking was carried out using the Discovery Studio 2019 Client software. We then set the chemical structure of the small-molecule compound as a ligand and added the CHARMM general force field. Discovery Studio 2019 Client was used to conduct the CDOCKER DOCK and save 2D and 3D visualization diagrams.

To detect the stability of the obtained ligand–receptor combinations in the molecular docking results, 50-ns molecular dynamics simulation of the protein–ligand complexes after molecular docking was conducted using Gromacs 2019.5 software. First, the GLO1-PCA file of the completed target protein was processed with PYMOL to form new proteins and small molecules, and then the related topological structure was generated. Then, the small-molecule compound substances were treated using Swiss Param. We added the information of small-molecule substances to the protein topology file to form a complex information file. Calculations were made using the CHARMM 36 force field and the TIP3P water model, and a regular 1.2-sized cube was set as the restricted box. We then added the solvent (SPC216 water) to it. The charge was balanced by adding Na/Cl ion pairs to the system. To avoid collision between proteins and small-molecule substances where possible, the fastest descent method was used to optimize the system to achieve a state of optimal potential energy. The regularization system was balanced at 300K, and the isothermal and isobaric system adopted Parrinelo–Rahman pressure coupling, which was carried out at 1bar (100°ps, dynamic step size 2 fs). Finally, after simulating the system for 50 ns, the molecular trajectories of the system were corrected for evaluation and calculation.

We then analyzed the root mean square deviation (RMSD), root mean square fluctuation (RMSF), radius of gyration (Rg), and solvent accessible surface area (SASA) in the molecular dynamics simulation trajectories of this group.

### 2.16 Statistical analysis

Statistical analysis was performed using GraphPad Prism 6.02 (GraphPad Software; La Jolla, CA, United States). Data were described as mean ± SD. Statistical comparisons were determined by one-way ANOVA followed by a Dunnett’s multiple comparisons test. Statistical significance was defined as p-value <0.05.

## 3 Results

### 3.1 Effect of PCA on the growth performance of rats with diabetic cataract

**FIGURE 1:**
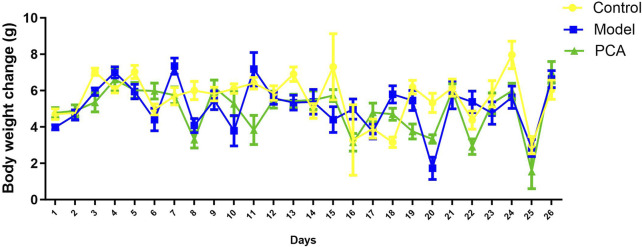
Effect of PCA (25 mg/kg) on body weight of diabetic cataract rat.

The effects of PCA on the growth performance of diabetic cataract were evaluated by measuring the body weight of the rats throughout the experimental period. As results shown in Figure 1, there was no significant difference in weight change among three groups. In other words, weight changes were similar across the groups. PCA had no significant effect on the body weight of rats.

### 3.2 Effect of PCA on the lens opacity of rat with diabetic cataract

**FIGURE 2:**
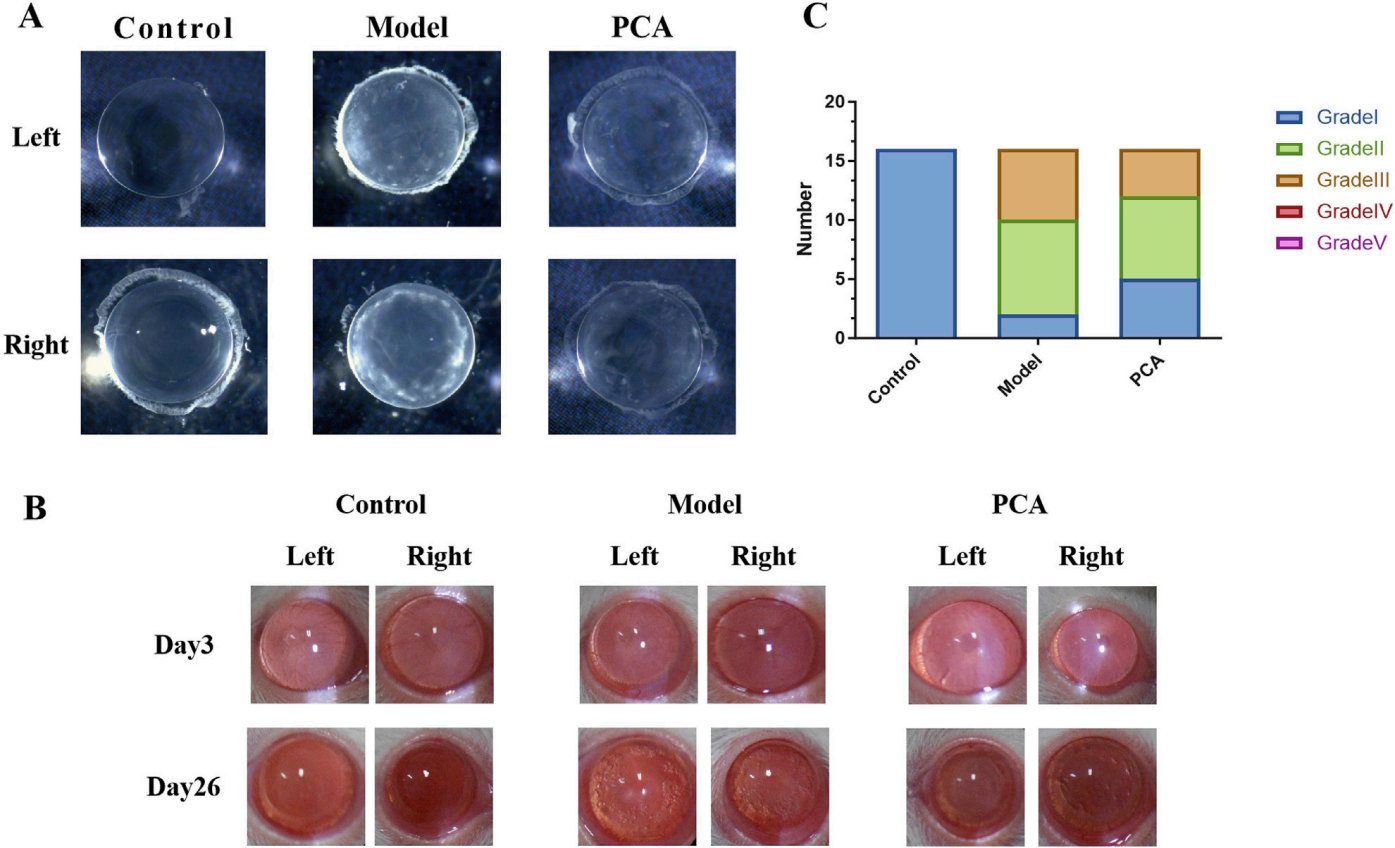
Effect of PCA (25 mg/kg) on lens opacity of diabetic cataract rats. **(A)** At the end of the experiment, the lens of the rat was placed in water, and its opacity was measured by ordinary microscope. **(B)** On days 3 and 26 of the experiment, the opacity of the rat lens was measured by slit-lamp microscopy. **(C)** At the end of the experiment, rat lens opacity was assessed according to the scoring criteria.

Lens opacity represents the progression of diabetic cataract, so this study evaluated the effect of PCA on lens opacity in diabetic cataract rats by slit-lamp examination. As shown in Figure 2A, according to the photographic results of an ordinary microscope, the opacity of the lens of rats in the model group compared with the control group was significantly increased at the end of the experiment; compared with the model group, the opacity of lens in the PCA group was significantly reduced. As shown in Figure 2B, slit-lamp examination was performed on rats in each group on the third day of the experiment, and the results showed that the lenses of each group were clear and transparent. On the 26th day of the experiment, compared with the control group, a large number of vacuoles appeared in the lens of rats in the model group, and the rate of vacuoles in the lens of rats in the PCA group was significantly reduced compared with that of the model group. In addition, at the end of the experiment, the rats of each group were scored for lens opacity. The results in Figure 2C show that all scores of the control group were Grade Ⅰ, and most scores of the model group were Grades Ⅱ and Ⅲ. In the PCA group, the proportion of Grades Ⅱ and Ⅲ decreased significantly, while the proportion of Grade Ⅰ increased significantly.

### 3.3 Effect of PCA on the pathological changes of the lens epithelium in rats with diabetic cataract

**FIGURE 3:**
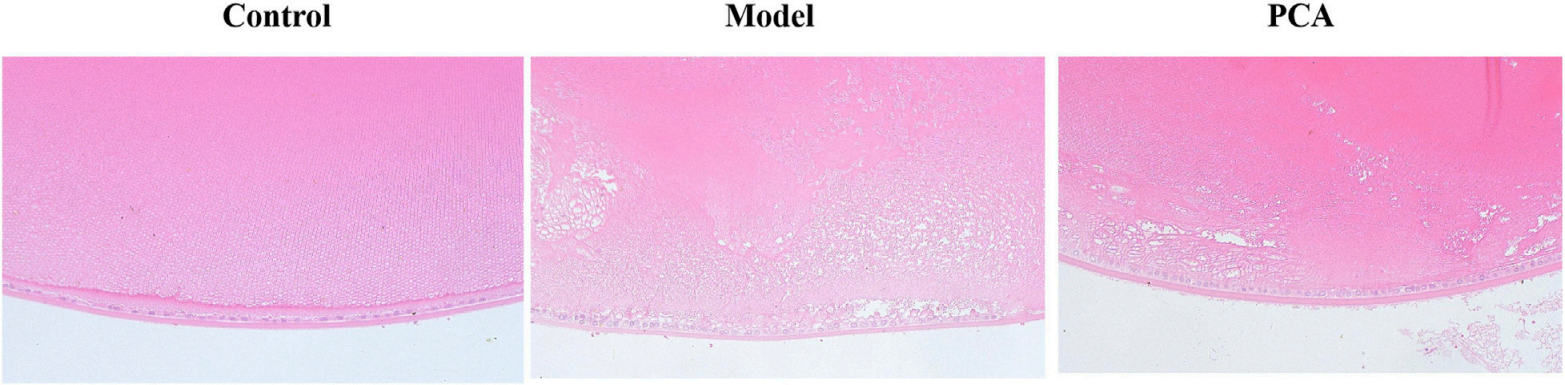
Effect of PCA (25 mg/kg) on the pathological changes of the lens epithelium in diabetic cataract rats by HE staining (original magnification ×20).

HE staining can be used to evaluate the pathological changes of DC rat lens epithelium—see Figure 3 for data. In the control group, the cells of the lens epithelium were arranged neatly and the shape was normal. Compared with the control group, the number of lens epithelial cells in the model group decreased, the arrangement was disordered, edema appeared, and cell space increased. Compared with the model group, the epithelial cells in the PCA group gradually increased, the cell edema reduced, and the epithelial cells were arranged in an orderly and tight manner.

### 3.4 Effect of PCA on the expression of GLO1 in rat with diabetic cataract

**FIGURE 4:**
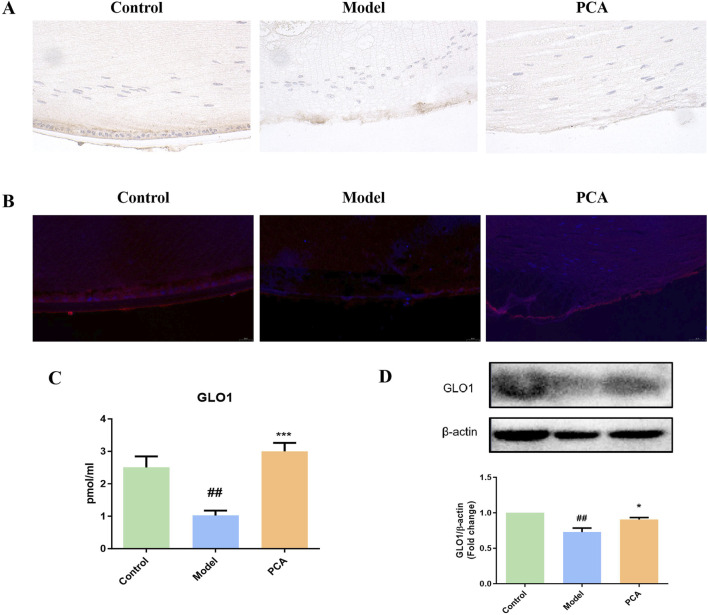
Effect of PCA (25 mg/kg) on the expression of GLO1 in diabetic cataract rats. **(A)** Expression of GLO1 in rat lens tissue by IHC staining (original magnification ×40). **(B)** Expression of GLO1 in rat lens tissue by IF staining (original magnification ×73). **(C)** Expression of GLO1 in rat lens tissue by ELISA. **(D)** Expression of GLO1 in rat lens tissue by WB. Data represented as mean ± SD. ^#^P < 0.05, ^##^P < 0.01 vs. control group; *P < 0.05, **P < 0.01 vs. model group. ^#^ for p < 0.05 between the model group and control group, ^##^ for p < 0.01 between the model group and control group, * for p < 0.05 between the PCA group and model group, and ** for p < 0.01 between the PCA group and model group.

GLO1 plays an important role in the regulation of glycosylation in diabetic cataract. In this part of the research, different methods were used to detect GLO1 expression. Based on the results of IHC and IF and compared with the control group, the number of GLO1 positive cells in the model group decreased significantly, while the number of GLO1-positive cells in the PCA group increased significantly compared with the model group (Figures 4A,B). According to the results of ELISA and Western blotting (WB), the expression of GLO1 protein in the lens of rats in the model group was significantly decreased compared with the control group, while the expression of GLO1 protein in the PCA group was significantly increased compared with the model group (Figures 4C,D).

### 3.5 Effect of PCA on the expression of AGEs in rat with diabetic cataract

**FIGURE 5:**
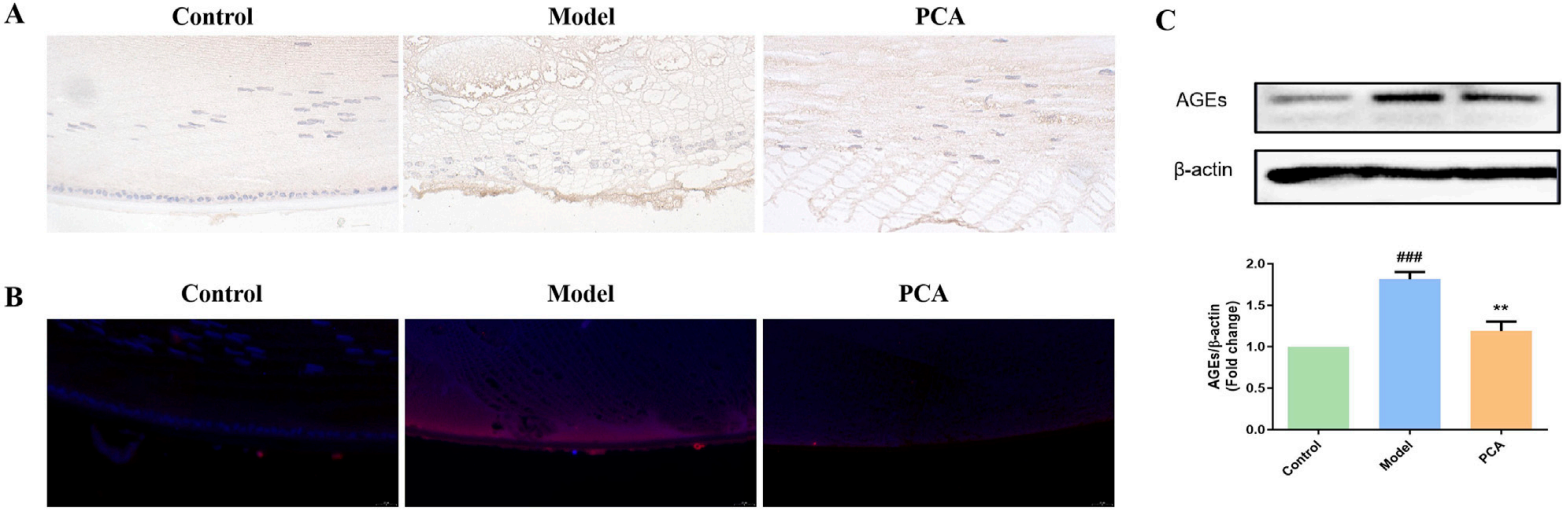
Effect of PCA (25 mg/kg) on the expression of AGEs in diabetic cataract rats. **(A)** Expression of AGEs in rat lens tissue by IHC staining (original magnification ×40). **(B)** Expression of AGEs in rat lens tissue by IF staining (original magnification ×73). **(C)** Expression of AGEs in rat lens tissue by WB. Data were represented as mean ± SD. ^#^P < 0.05, ^##^P < 0.01 vs. control group; *P < 0.05, **P < 0.01 vs. model group. ^#^ for p < 0.05 between the model group and control group, ^##^ for p < 0.01 between the model group and control group, * for p < 0.05 between the PCA group and model group, and ** for p < 0.01 between the PCA group and model group.

AGEs represent the progression of glycosylation in diabetic cataract. According to the results of IHC and IF, the number of AGE-positive cells in the model group increased significantly compared with the control group, while the number of AGE-positive cells in the PCA group decreased significantly compared with the model group (Figures 5A,B). According to the results of WB, the expression of AGE protein in the lens of rats in the model group was significantly increased compared with that in the control group, while the expression of AGE protein in the PCA group was significantly decreased compared with that in the model group (Figure 5C).

### 3.6 Effect of PCA on the expression of RAGE in rat with diabetic cataract

**FIGURE 6:**
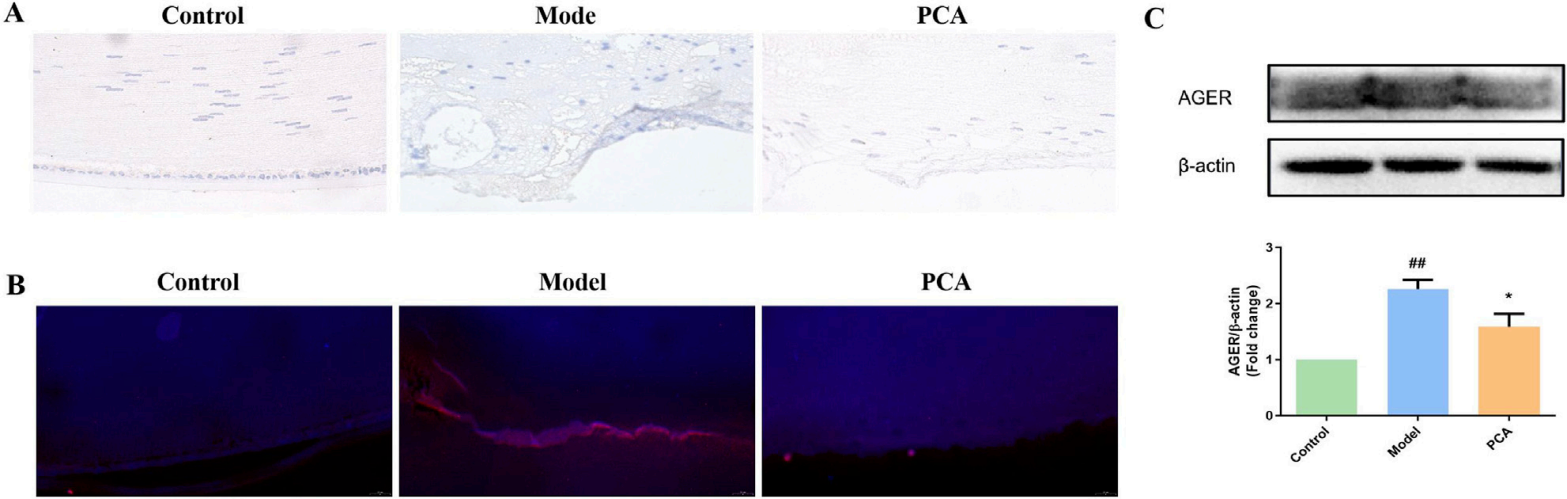
Effect of PCA (25 mg/kg) on expression of RAGE in diabetic cataract rats. **(A)** Expression of RAGE in rat lens tissue by IHC staining (original magnification ×40). **(B)** Expression of RAGE in rat lens tissue by IF staining (original magnification ×73). **(C)** Expression of RAGE in rat lens tissue by WB. Data represented as mean ± SD. ^#^P < 0.05, ^##^P < 0.01 vs. control group; *P < 0.05, **P < 0.01 vs. model group. ^#^ for p < 0.05 between the model group and control group, ^##^ for p < 0.01 between the model group and control group, * for p < 0.05 between the PCA group and model group, and ** for p < 0.01 between the PCA group and model group.

RAGE also represents the progression of glycosylation in diabetic cataract. According to the results of IHC and IF, the number of RAGE-positive cells in the model group increased significantly compared with that in the control group, while the number of RAGE-positive cells in the PCA group decreased significantly compared with that in the model group (Figures 6A,B). According to the results of WB, the expression of RAGE protein in the lens of rats in the model group was significantly increased compared with that in the control group, while the expression of RAGE protein in the PCA group was significantly decreased compared with that in the model group (Figure 6C).

### 3.7 Effect of PCA on the oxidative stress response in rat with diabetic cataract

**FIGURE 7:**
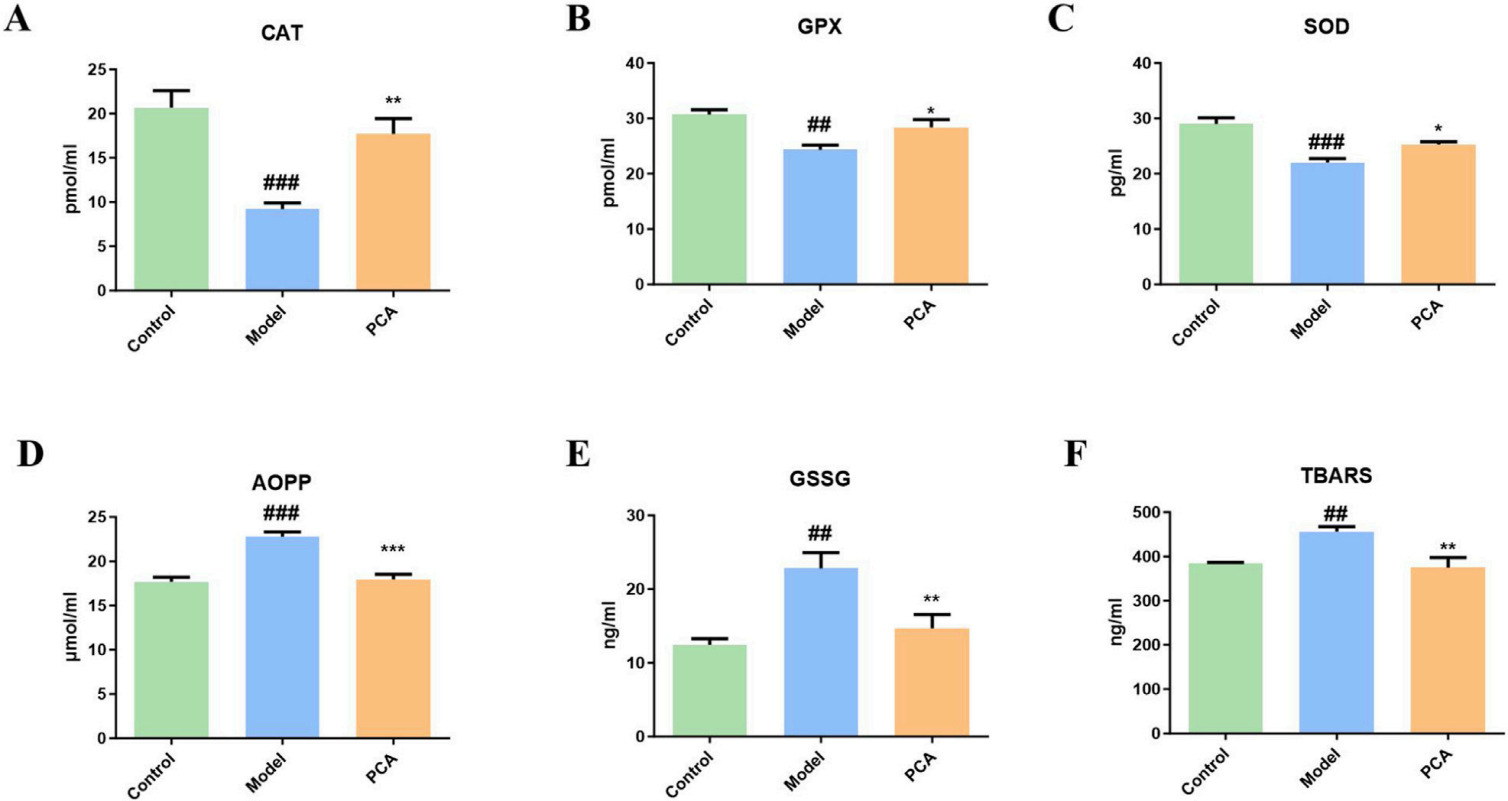
Effect of PCA (25 mg/kg) on oxidative stress response in diabetic cataract rats. **(A)** Level of CAT in rat lens tissue by ELISA. **(B)** Level of GPX in rat lens tissue by ELISA. **(C)** Level of SOD in rat lens tissue by ELISA. **(D)** Level of AOPP in rat lens tissue by ELISA. **(E)** Level of GSSG in rat lens tissue by ELISA. **(F)** Level of TBARS in rat lens tissue by ELISA. Data represented as mean ± SD. ^#^P < 0.05, ^##^P < 0.01 vs. control group; *P < 0.05, **P < 0.01 vs. model group. ^#^ for p < 0.05 between the model group and control group, ^##^ for p < 0.01 between the model group and control group, * for p < 0.05 between the PCA group and model group, and ** for p < 0.01 between the PCA group and model group.

Glycosylation aggravates oxidative stress in diabetic cataract. This partly reflects the progression of oxidative stress through the detection of markers by ELISA. In Figure 7, compared with the control group, the expressions of CAT, GPX, and SOD in the model group were significantly reduced, while the expressions of CAT, GPX, and SOD in the PCA group were significantly increased compared with the model group. In addition, compared with the control group, the expression of oxidative stress markers AOPP, GSSG, and TBARS in the model group were significantly increased, while the expressions of CAT, GPX, and SOD in the PCA group were significantly decreased compared with the model group.

### 3.8 Effect of PCA on the cell viability of human lens epithelial cells

**FIGURE 8:**
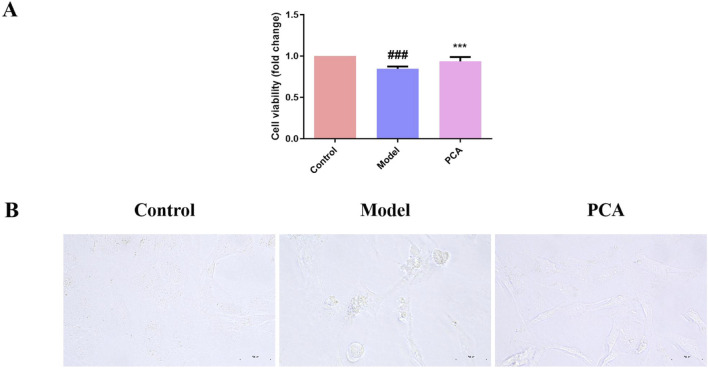
Effect of PCA (10 μM) on cell viability in human lens epithelial cells. **(A)** Cell viability of human lens epithelial cells measured by CCK-8 kit. **(B)** Morphology of human lens epithelial cells photographed with microscope (original magnification ×40). Data represented as mean ± SD. ^#^P < 0.05, ^##^P < 0.01 vs. control group; *P < 0.05, **P < 0.01 vs. model group. ^#^ for p < 0.05 between the model group and control group, ^##^ for p < 0.01 between the model group and control group, * for p < 0.05 between the PCA group and model group, and ** for p < 0.01 between the PCA group and model group.

The effect of PCA on the DC cell model was examined by stimulating lens epithelial cells with high glucose. In Figure 8, compared with the control group, the cell viability in the model group was significantly reduced, and the cell morphology was also changed under the microscope. Compared with the model group, the cell viability in the PCA group was significantly increased, and cell morphology gradually returned to normal under the microscope.

### 3.9 Effect of PCA on the expression of GLO1 in human lens epithelial cells

**FIGURE 9:**
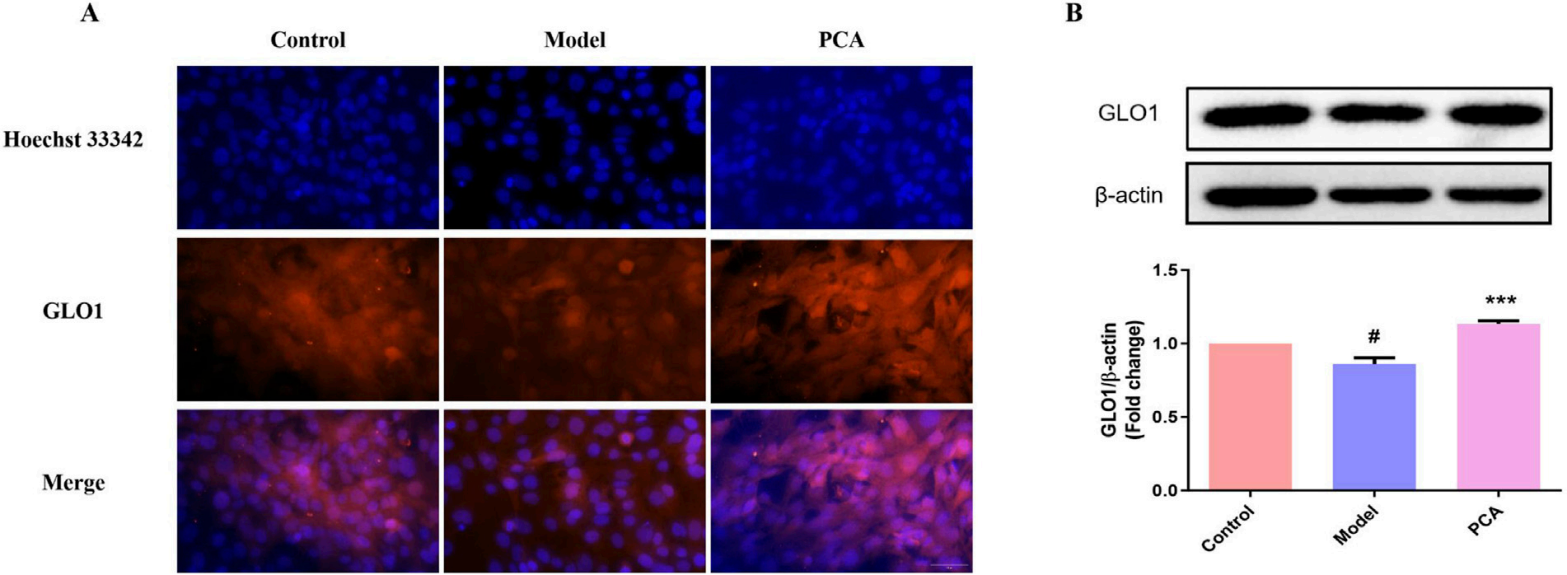
Effect of PCA (10 μM) on expression of GLO1 in human lens epithelial cells. **(A)** Expression of GLO1 in human lens epithelial cells by IF staining (original magnification ×40). **(B)** Expression of GLO1 in human lens epithelial cells by WB. Data represented as mean ± SD. ^#^P < 0.05, ^##^P < 0.01 vs. control group; *P < 0.05, **P < 0.01 vs. model group. ^#^ for p < 0.05 between the model group and control group, ^##^ for p < 0.01 between the model group and control group, * for p < 0.05 between the PCA group and model group, and ** for p < 0.01 between the PCA group and model group.

GLO1 was also examined in the cell model. Based on the results of IF, the number of GLO1-positive cells in the model group decreased significantly compared with the control group, while the number of GLO1-positive cells in the PCA group increased significantly compared with the model group (Figure 9A). In WB results, the expression of GLO1 protein in human lens epithelial cells in the model group was significantly decreased compared with the control group, while the expression of GLO1 protein in the PCA group was significantly increased compared with the model group (Figure 9B).

### 3.10 Effect of PCA on the expression of AGEs in human lens epithelial cells

**FIGURE 10:**
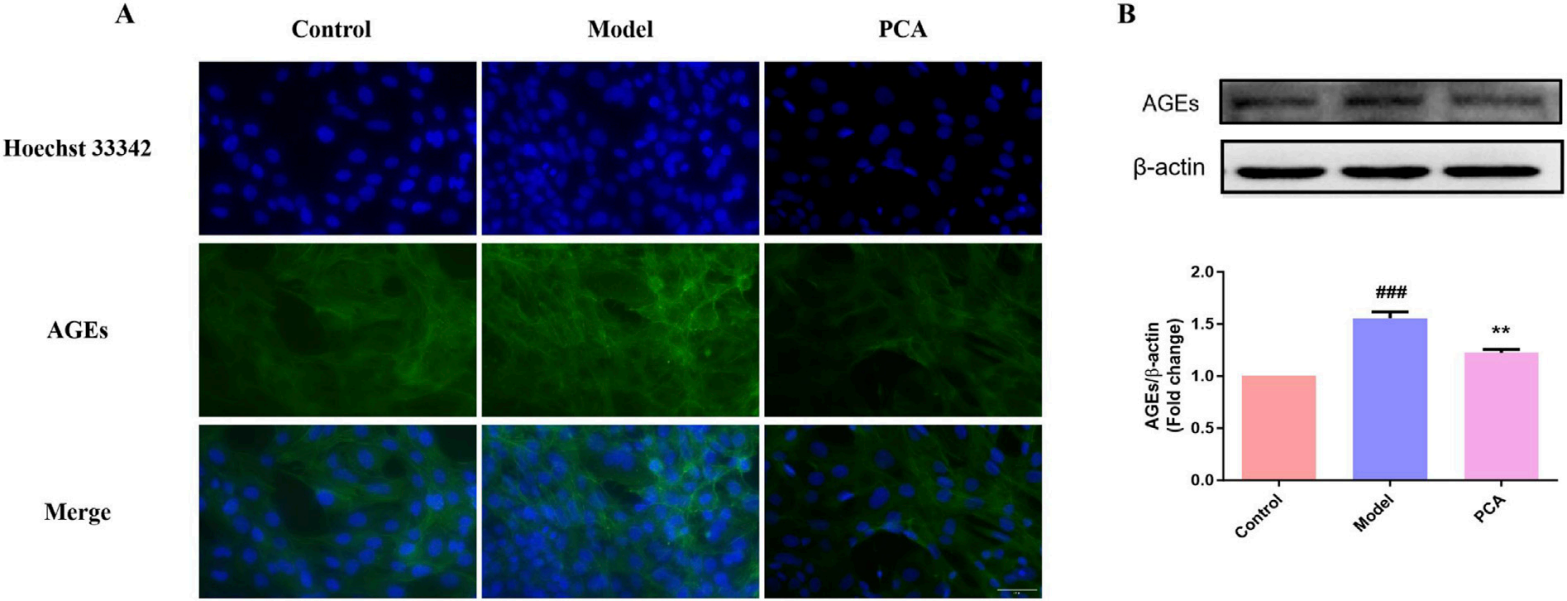
Effect of PCA (10 μM) on expression of AGEs in human lens epithelial cells. **(A)** Expression of AGEs in human lens epithelial cells by IF staining (original magnification ×40). **(B)** Expression of AGEs in human lens epithelial cells by WB. Data represented as mean ± SD. ^#^P < 0.05, ^##^P < 0.01 vs. control group; *P < 0.05, **P < 0.01 vs. model group. ^#^ for p < 0.05 between the model group and control group, ^##^ for p < 0.01 between the model group and control group, * for p < 0.05 between the PCA group and model group, and ** for p < 0.01 between the PCA group and model group.

AGEs were also examined in the cell model. According to the results of IF, the number of AGE-positive cells in the model group increased significantly compared with the control group, while the number of AGE-positive cells in the PCA group decreased significantly compared with the model group (Figure 10A). According to the results of WB, the expression of AGE protein in human lens epithelial cells in the model group was significantly increased compared with the control group, while the expression of AGE protein in the PCA group was significantly decreased compared with the model group (Figure 10B).

### 3.11 Effect of PCA on the expression of RAGE in human lens epithelial cells

**FIGURE 11:**
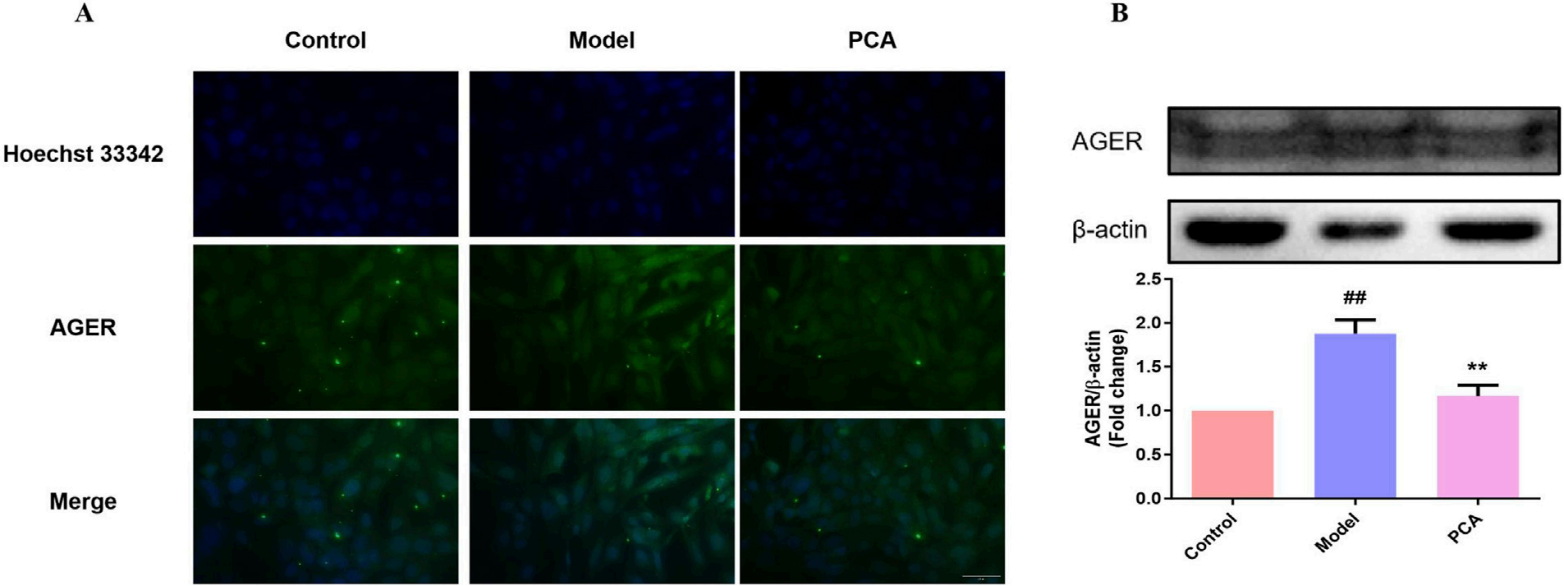
Effect of PCA (10 μM) on expression of RAGE in human lens epithelial cells. **(A)** Expression of RAGE in human lens epithelial cells by IF staining (original magnification ×40). **(B)** Expression of RAGE in human lens epithelial cells by WB. Data represented as mean ± SD. ^#^P < 0.05, ^##^P < 0.01 vs. control group; *P < 0.05, **P < 0.01 vs. model group. ^#^ for p < 0.05 between the model group and control group, ^##^ for p < 0.01 between the model group and control group, * for p < 0.05 between the PCA group and model group, and ** for p < 0.01 between the PCA group and model group.

RAGE was also examined in the cell model. In IF results, the number of RAGE-positive cells in the model group increased significantly compared with the control group, while the number of RAGE-positive cells in the PCA group decreased significantly compared with the model group (Figure 11A). According to the results of WB, the expression of RAGE protein in human lens epithelial cells in the model group was significantly increased compared with the control group, while the expression of RAGE protein in the PCA group was significantly decreased compared with the model group (Figure 11B).

### 3.12 Effect of PCA on the oxidative stress response of human lens epithelial cells

**FIGURE 12:**
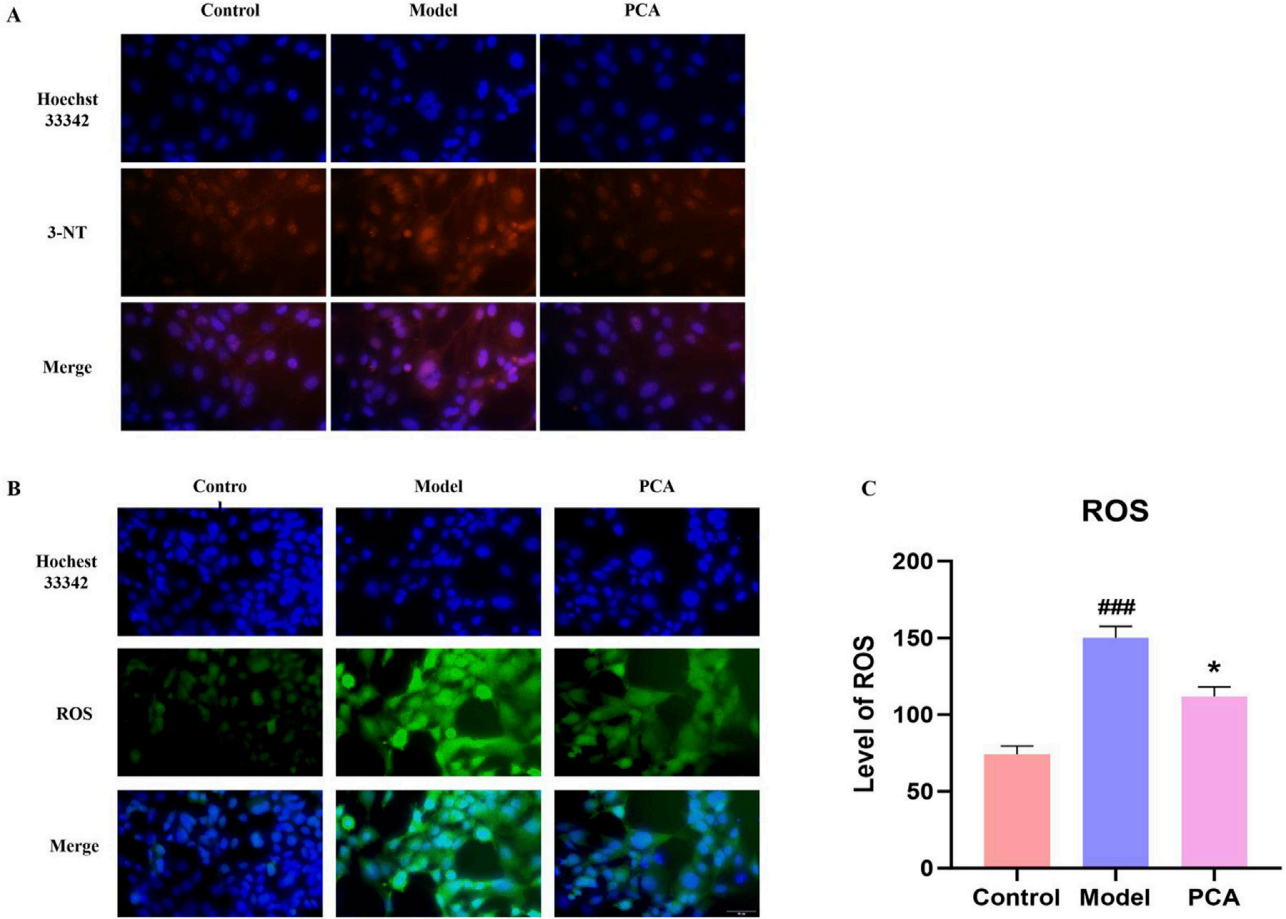
Effect of PCA (10 μM) on oxidative stress response in human lens epithelial cells. **(A)** Expression of 3-NT in human lens epithelial cells by IF staining (original magnification ×40). **(B, C)** Level of ROS in human lens epithelial cells by ROS staining (original magnification ×40). Data represented as mean ± SD. ^#^P < 0.05, ^##^P < 0.01 vs. control group; *P < 0.05, **P < 0.01 vs. model group. ^#^ for p < 0.05 between the model group and control group, ^##^ for p < 0.01 between the model group and control group, * for p < 0.05 between the PCA group and model group and, ** for p < 0.01 between the PCA group and model group.

The progression of oxidative stress was also examined in a diabetic cataract cell model. According to the results of IF, the number of positive cells of the oxidative stress marker 3-NT in the model group was significantly increased compared with the control group, and the number of positive cells of 3-NT was significantly decreased compared with the model group (Figure 12A). According to the fluorescence results of ROS, the number of ROS-positive cells in the model group was significantly increased compared with the control group, and the number of ROS-positive cells was significantly decreased compared with the model group (Figures 12B,C).

### 3.13 Molecular docking of PCA and GLO1

**FIGURE 13:**
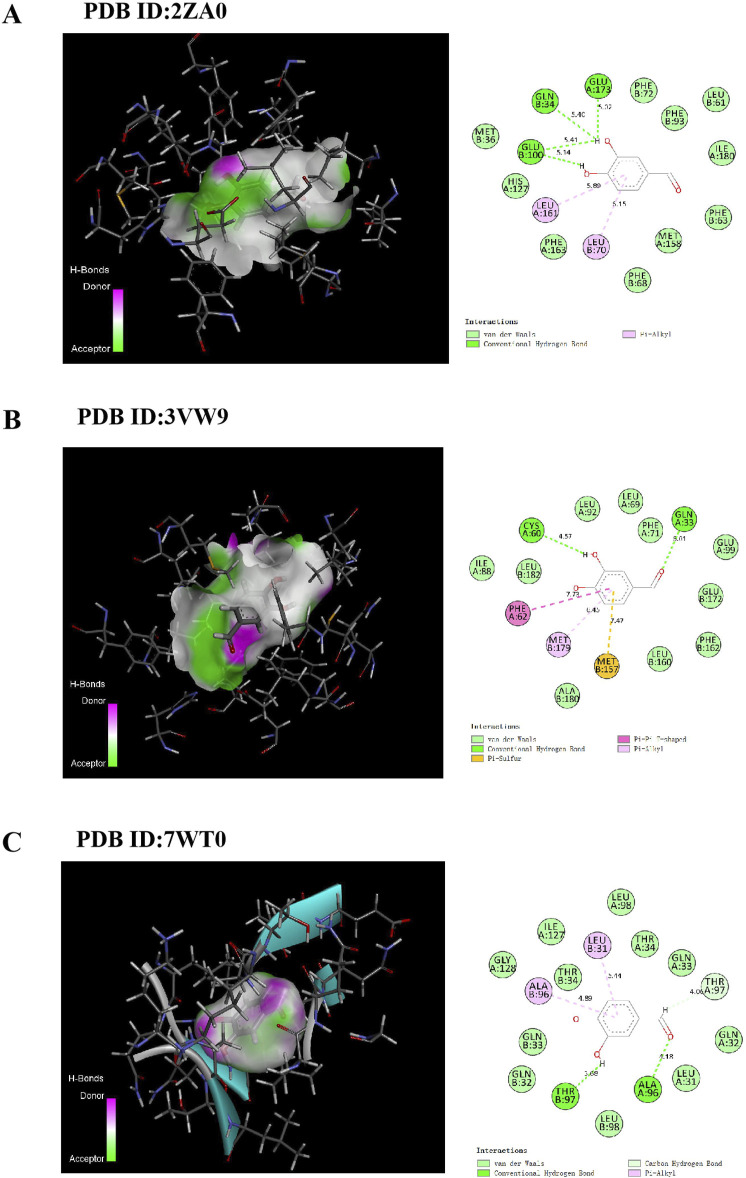
Docking patterns of PCA interacting with 2ZA0 **(A)**, 3VW9 **(B)** and 7WT0 **(C)** as produced using CDOCKER.

Molecular docking was performed to investigate potential binding modes and interactions between PCA and GLO1. The results of PCA docking with three GLO1 protein molecules with different structures are as follows: PCA likely interacts strongly with GLO1, as suggested through -CDOCKER energies of 26.4065, 24.3121, and 24.3176 (Table 1); the -CDOCKER ENERGY of PCA and 2ZA0 complex is the highest (26.4065). In addition, the binding energy of PCA and 3VW9 complex is the highest (94.8852 kcal/mol). The results suggest that PCA produces conventional hydrogen bonds with GLN34, GLU173, and GLU100 of 2ZA0; van der Waals interactions with MET36, PHE72, PHE93, LEU61, ILE180, PHE63, MET158, PHE68, PHE163, and HIS127; and pi–alkyl interactions with LEU161 and LEU70 (Figure 13A). According to the docking result of 3VW9, there are not only conventional hydrogen bonds with CYS60 and GLN33 and a pi–pi T-shaped interaction with PHE62 but also a pi–sulfur interaction with MET157 and a pi–alkyl interaction with MET179 (Figure 13B). The interaction with 7WT0 includes conventional hydrogen bonds with THR97 and ALA96, a carbon hydrogen bond with THR97, and pi–alkyl interactions with LEU31 and ALA96 (Figure 13C).

**TABLE 1:** CDOCKER analysis of PCA and GLO1.

Protein	Binding energy (kcal/mol)	-Cdocker energy	-Cdocker interaction energy
p-2za0 complex	36.0935	26.4065	26.8543
p-3vw9 complex	94.8852	24.3121	25.3838
p-7wt0 complex	−0.8771	24.3176	25.6864

### 3.14 Molecular dynamics simulation of PCA and GLO1

**FIGURE 14:**
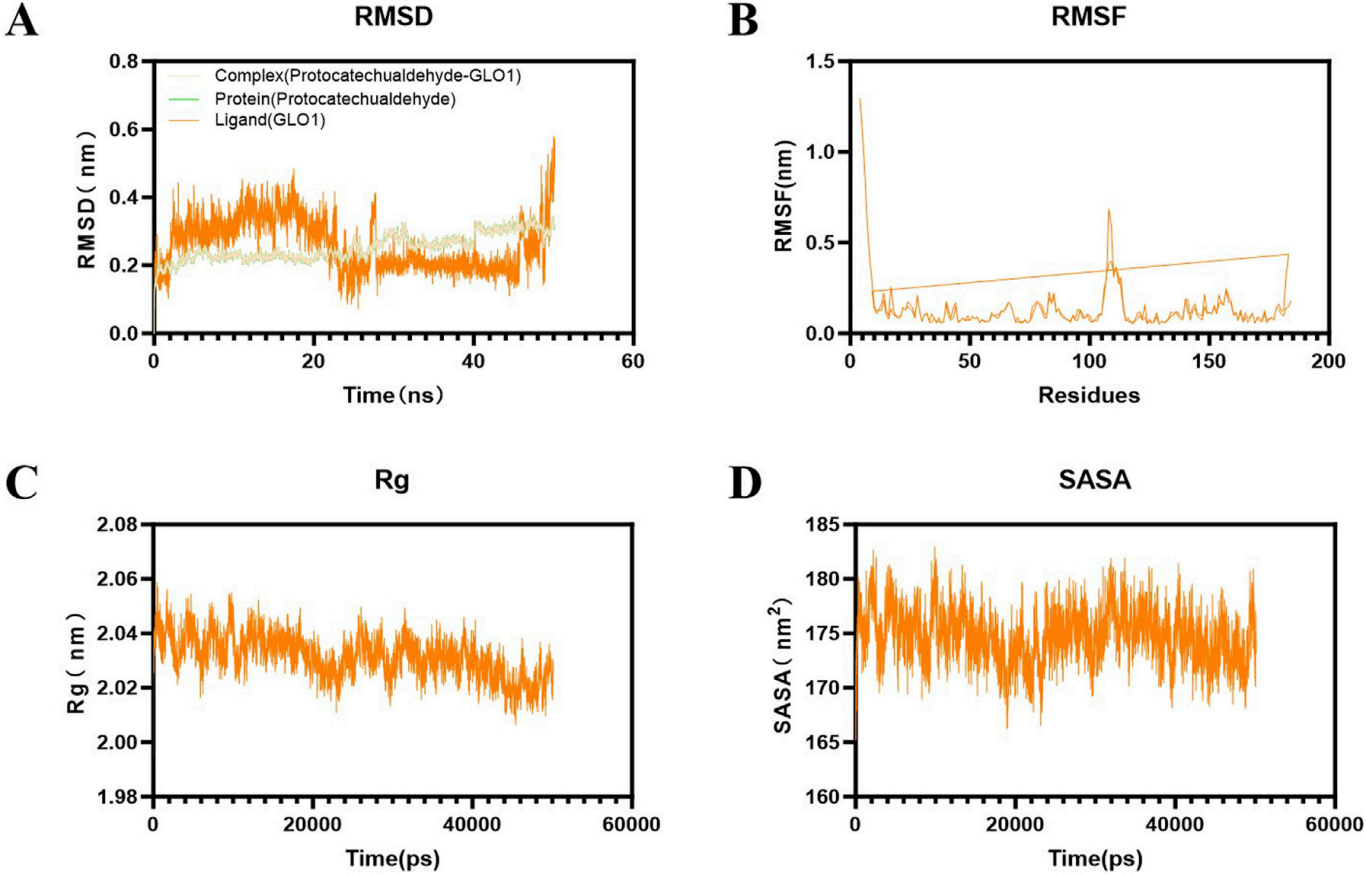
Results of molecular dynamics simulation of PCA and GLO1. **(A)** Result of RMSD between PCA and GLO1. **(B)** Result of RMSF between PCA and GLO1. **(C)** Result of Rg between PCA and GLO1. **(D)** Result of SASA between PCA and GLO1.

RMSD is typically used to describe the conformational changes of proteins or molecular ligands in molecular simulations. The RMSD values of the system are mostly between 0.2 and 0.4 nm, and the overall structure is relatively stable. The RMSD of the protein remains consistent with that of the whole, while the RMSD of the ligand is still changing and has not yet reached stability. The results show that during the entire simulation period, the RMSD value of the system was small, indicating that the system deviation is small and no significant conformational changes occur in the molecules (Figure 14A). RMSF can be used to describe the range of motion of each atom in the protein structure in molecular simulation. During the protein molecule simulation process, protein molecules underwent a certain degree of vibration and oscillation. RMSF can calculate the intensity and direction of these vibrations, thereby characterizing the flexibility and movement intensity of each amino acid in the protein throughout the simulation process. Analysis shows that the fluctuation of RMSF during the entire simulation process was relatively small, generally within the range of 0.1–0.3 nm. In the 105–115 region, RMSF varies greatly, which may be related to the protein adjustment conformation (Figure 14B).

Rg can characterize the protein structure’s compactness. The results show that its radius generally has a downward trend throughout the simulation process, indicating that its protein structure gradually becomes more compact (Figure 14C). SASA can characterize the change in the surface area of the solution that ligands can directly contact over time, reflecting the relative exposure degree of ligands in the solvent and thereby indirectly reflecting the tightness of protein encapsulation of ligands. The results show that the SASA of the complex system is generally stable, mostly between 170 and 180 nm^2^ (Figure 14D).

## 4 Discussion

PCA is the main component of a variety of Chinese herbs (such as *S. miltiorrhiza* and *Gastrodia elata*) which have many advantages, such as low toxicity, fast metabolic rate, and few adverse reactions (Anwar et al., 2021; Cheng et al., 2021). *In vivo* and *in vitro* experiments have proven that PCA has many pharmacological effects such as anti-inflammatory (Wei et al., 2013; Zhang et al., 2024), antioxidant (Chang et al., 2011), inhibition of apoptosis (Xing et al., 2012), and anti-fibrosis (Semaming et al., 2015). It has also been proven to effectively improve the progression of STZ-induced diabetic cataract (Ji et al., 2021). However, there is little study of the mechanism of PCA in diabetic cataract.

In this study, galactose was used to construct a diabetic cataract rat model. The animal experiment results showed that PCA had no significant effect on the body weight of diabetic cataract rats, but in the slit-lamp test, PCA could significantly improve the lens opacity of diabetic cataract rats, reduce the lens vacuole, and significantly reduce the lens opacity score. In addition, HE staining results also showed that PCA gradually increased the number of lens epithelial cells and arranged them closely, and the cell edema was reduced. This is consistent with previous research (Kim et al., 2007). In addition, a diabetic cataract cell model was constructed by stimulation of human lens epithelial cells with high glucose. The cell experiment results showed that PCA could significantly increase the cell viability and improve cell morphology so that the cells could return to normal. This is also consistent with the results of previous studies (Kim et al., 2007). Overall, the results of the above pharmacodynamic experiments suggest that PCA can indeed improve the progression of diabetic cataract and the opacity of the cataract lens.

The pathogenesis of DC is very complex, including the activation of AR receptors, accumulation of AGEs, and oxidative stress damage caused by ROS (Hilliard et al., 2020; Liu et al., 2020; Armao et al., 2019; Tao et al., 2022). Among these, glycosylation and oxidative stress play key roles in the pathogenesis of DC. Non-enzymatic glycosylation is a complex cascade reaction (Zych et al., 2020; Antonetti et al., 2021). AGEs are glycosylated products formed by non-enzymatic reactions with amino groups of large biomolecules stimulated by chronic hyperglycemia (Dehnad et al., 2020). Emerging evidence underscores the pivotal role of AGE/RAGE activation in cataract progression, particularly through glycative and oxidative stress pathways (Ruiz et al., 2020; Truong et al., 2019). In diabetic models, AGEs accumulate in the lens due to hyperglycemia-driven non-enzymatic glycation, forming covalent cross-links with crystallin proteins. This disrupts lens transparency by altering crystallin tertiary structures and promoting light scattering, a hallmark of age-related nuclear cataracts (Yun, 2020; Jiang et al., 2022). Furthermore, RAGE activation in LECs triggers NF-κB-mediated inflammation and NLRP3 inflammasome signaling, amplifying oxidative stress and apoptosis. For instance, in diabetic cataract models, RAGE overexpression correlates with elevated ROS production and caspase-3 activation, driving LEC death and exacerbating lens opacification (Dalal et al., 2025; Zhang et al., 2024b). AGEs also impair the blood–aqueous barrier, facilitating intraocular inflammatory cytokine infiltration, which accelerates PCO via TGF-β-/Smad-dependent EMT in residual LECs (Yun, 2020; Bai et al., 2024). Notably, interventions targeting AGEs/RAGE, such as GLO1 upregulation or RAGE antagonists, have shown promise in reducing lens glycation and preserving transparency in preclinical studies (Jiang et al., 2022; Dalal et al., 2025). These findings align with broader observations in AGE-related pathologies, such as diabetic nephropathy and osteoarthritis, where RAGE inhibition mitigates oxidative damage and tissue fibrosis (Jiang et al., 2022; Yibo, 2023). Together, these mechanisms position AGEs/RAGE as a central therapeutic target for cataracts, particularly in diabetic and aging populations. Both animal experiments and cell experiments in this study showed that the levels of AGEs in diabetic cataract were significantly increased by IHC, IF, and WB detection, while PCA could significantly decrease the expression of AGEs, which was consistent with Ji et al. (2021) and Wang et al. (2014). In addition to being deposited in the extracellular matrix, AGEs also bind to RAGE and activate Nox and NF-κB (Pang et al., 2020; Chaudhuri et al., 2018), thus initiating a vicious cycle of oxidative stress and inflammation (Wang et al., 2019; Kopytek et al., 2020). While this study did not directly quantify Nox4 or p-NF-κB, future studies incorporating Nox4 inhibition or p-NF-κB immunostaining will further delineate this pathway. In the animal and cell experiments of this study, it was found that the level of RAGE in diabetic cataract is also significantly increased through IHC, IF, and WB detection, while PCA could significantly reduce the expression of RAGE, thus reducing the degree of glycosylation. In general, PCA slows the progression of glycosylation in diabetic cataracts by reducing AGEs/RAGE levels.

Since the formation of AGEs cannot be reversed, their accumulation in the lens continues to cause oxidative stress, even if high blood sugar is improved, which in turn further exacerbates disease progression (Bheda, 2020). The following results of this study suggest that oxidative stress is indeed involved in the progression of diabetic cataract. In diabetic cataract rats, the levels of the antioxidant enzymes CAT, GPX, and SOD were significantly decreased, while the expressions of the oxidative stress markers AOPP, GSSG, and TBARS were significantly decreased. Overall, the decrease of antioxidant enzymes and the increase of oxidative stress products suggest that the progression of glycosylation in diabetic cataract is accompanied by intense oxidative stress. In addition, at the cellular level, the study showed through the detection of ROS probe and 3-NT immunofluorescence stain, a protein nitration marker, that the levels of ROS and 3-NT in human lens epithelial cells were also significantly increased under the stimulation of high sugar, which was the same conclusion as the animal experiments. PCA has the parent nuclear structure of o-diphenol, which is the material basis of its significant antioxidant activity. Studies on the correlation between the composition of *S. miltiorrhiza* and antioxidant activity showed that the antioxidant activity of PCA in scavenging 1, 1-diphenyl-picrohydrazyl (DPPH) free radicals was stronger than that of danshensu sodium and caffeic acid (Wu et al., 2009). In this study, it was first found by ELISA that PCA can increase the levels of antioxidant enzymes CAT, GPX, and SOD and decrease the levels of oxidative stress markers AOPP, GSSG, and TBARS in diabetic cataract rats. Then, it was found by ROS probe that PCA can reduce ROS levels in diabetic cataract cell models. Finally, it was found by IF that PCA could reduce the expression of 3-NT, a marker of oxidative stress, in the cell model. In summary, glycosylation increases the progression of oxidative stress in diabetic cataract, while PCA improves oxidative stress by reducing glycosylation.

AGE-RAGE signaling is mainly regulated by the upstream factor glyoxalase system, and GLO1 plays an important role as a rate-limiting enzyme in this regulatory system. Physiologically, GLO1 can degrade the main precursor of AGEs (Sun et al., 2020). However, in chronic hyperglycemia, the function and expression of GLO1 are reduced, which intensifies the accumulation of AGEs and the pathological activation of AGE-RAGE signaling (Rabbani and Thornalley, 2018; Rabbani et al., 2022). This is the same as the results of this study. In this research, the level of GLO1 in the lens of diabetic cataract rats was significantly decreased by the detection of IHC, IF, ELISA, and WB, and the expression of GLO1 in the human lens epithelial cells stimulated by high glucose was also significantly decreased by the detection of IF and ELISA. Therefore, the search for drugs that can enhance the expression and function of GLO1 has great benefits for the treatment of AGE-RAGE glycosylation-mediated DC oxidative stress. In this study, both animal and cell experiments have proved that PCA can significantly increase the expression of GLO1 and improve the progression of glycosylation in diabetic cataract. While these data demonstrate that PCA significantly upregulates GLO1 expression, the precise regulatory mechanism warrants further investigation. GLO1 induction could involve transcriptional activation via stress-responsive pathways such as the Nrf2/ARE axis, which is known to regulate antioxidant and detoxification enzymes in diabetic models (Zhu et al., 2020). Alternatively, PCA may enhance GLO1 protein stability or directly modulate its enzymatic activity, as seen with other polyphenols like resveratrol in AGE-related pathways (Li et al., 2023). For instance, quercetin, a structurally analogous flavonoid, was shown to stabilize GLO1 mRNA and protein levels under hyperglycemic conditions, suggesting a potential post-transcriptional mechanism (Liu et al., 2019). Future studies employing qPCR to quantify GLO1 mRNA levels, cycloheximide chase assays to assess protein half-life, and enzymatic activity assays under PCA treatment would clarify these possibilities. Such mechanistic insights would not only validate these findings but also inform the development of PCA-based therapies that target GLO1 in diabetic complications. Furthermore, molecular docking techniques are used in this article to discuss the specific docking modes of PCA and GLO1 and explore whether PCA directly affects the expression of GLO1 through molecular docking with GLO1. Three different crystal structures of GLO1 were selected for molecular docking. From the docking results, PCA likely interacts strongly with GLO1. Moreover, in this research, the stability of the molecular structures of PCA and GLO1 is evaluated through molecular dynamics simulation experiments. It can be determined from the simulation results of RMSD, RNSF, Rg, and SASA that the molecular conformations of PCA and GLO1 are relatively stable. This suggests that PCA is likely to have direct molecular interactions with GLO1 to regulate glycosylation, thereby ameliorating oxidative stress in diabetic cataract; however, further research is needed to confirm this. The limitations of this study also include the lack of validation experiments to confirm the docking results, which is a future research direction of this study.

## 5 Conclusion

In this study, a rat model of diabetic cataract was established by galactose, and a cell model of diabetic cataract was established by stimulation of human lens epithelial cells with high glucose. From the animal, cellular, and molecular levels, the mechanism of PCA to improve oxidative stress in diabetic cataract by regulating GLO1 to inhibit AGE-RAGE glycosylation was verified. This lays a solid foundation for the drug development of diabetic cataract. However, there are still some shortcomings in this study. For example, the expression of AGE-RAGE and the downstream oxidative stress level need to be verified through inhibition and amplification of GLO1 expression. In addition, the interaction site of PCA and GLO1 needs demonstrating *in vivo* to confirm the interaction relationship between the two. These will be further studied in subsequent research.

## Data Availability

The original contributions presented in the study are included in the article/Supplementary Material; further inquiries can be directed to the corresponding authors.

## References

[B1] Antonetti D. A. Silva P. S. Stitt A. W. (2021). Current understanding of the molecular and cellular pathology of diabetic retinopathy. Nat. Rev. Endocrinol. 17 (4), 195–206. 10.1038/s41574-020-00451-4 33469209 PMC9053333

[B2] Anwar S. Khan S. Almatroudi A. Khan A. A. Alsahli M. A. Almatroodi S. A. (2021). A review on mechanism of inhibition of advanced glycation end products formation by plant derived polyphenolic compounds. Mol. Biol. Rep. 48 (1), 787–805. 10.1007/s11033-020-06084-0 33389535

[B3] Armao D. Bouldin T. W. Bailey R. M. Hooper J. E. Bharucha D. X. Gray S. J. (2019). Advancing the pathologic phenotype of giant axonal neuropathy: early involvement of the ocular lens. Orphanet J. Rare Dis. 14 (1), 27. 10.1186/s13023-018-0957-5 30709364 PMC6359799

[B4] Bai L. Feng M. Zhang Q. Cai Z. Li Q. Li Y. (2024). Synergistic osteogenic and antiapoptotic framework nucleic acid complexes prevent diabetic osteoporosis. Adv. Funct. Mater. 34, 2314789. 10.1002/adfm.202314789

[B5] Bheda P. (2020). Metabolic transcriptional memory. Mol. Metab. 38, 100955. 10.1016/j.molmet.2020.01.019 32240621 PMC7300383

[B6] Brownlee M. (2001). Biochemistry and molecular cell biology of diabetic complications. Nature 414 (6865), 813–820. 10.1038/414813a 11742414

[B7] Chang Z. Q. Gebru E. Lee S. P. Rhee M. H. Kim J. C. Cheng H. (2011). *In vitro* antioxidant and anti-inflammatory activities of protocatechualdehyde isolated from Phellinus gilvus. J. Nutr. Sci. Vitaminol. (Tokyo) 57 (1), 118–122. 10.3177/jnsv.57.118 21512301

[B8] Chaudhuri J. Bains Y. Guha S. Kahn A. Hall D. Bose N. (2018). The role of advanced glycation end products in aging and metabolic diseases: bridging association and causality. Cell Metab. 28 (3), 337–352. 10.1016/j.cmet.2018.08.014 30184484 PMC6355252

[B9] Cheng X. Song Z. Wang X. Xu S. Dong L. Bai J. (2021). A network pharmacology study on the molecular mechanism of protocatechualdehyde in the treatment of diabetic cataract. Drug Des. Devel Ther. 15, 4011–4023. 10.2147/DDDT.S334693 PMC8476343 34594100

[B10] Dalal D. Singh L. Singh A. (2025). Calycosin and kidney health: a molecular perspective on its protective mechanisms. Pharmacol. Rep. 77 (3), 658–669. 10.1007/s43440-025-00728-3 40249500

[B11] Dehnad A. Fan W. Jiang J. X. Fish S. R. Li Y. Das S. (2020). AGER1 downregulation associates with fibrosis in nonalcoholic steatohepatitis and type 2 diabetes. J. Clin. Invest 130 (8), 4320–4330. 10.1172/JCI133051 32657776 PMC7410084

[B12] Ding H. Liu J. Chen Z. Huang S. Yan C. Kwek E. (2024). Protocatechuic acid alleviates TMAO-aggravated atherosclerosis via mitigating inflammation, regulating lipid metabolism, and reshaping gut microbiota. Food Funct. 15 (2), 881–893. 10.1039/d3fo04396g 38165856

[B13] Erukainure O. L. Hafizur R. M. Choudhary M. I. Adhikari A. Mesaik A. M. Atolani O. (2017). Anti-diabetic effect of the ethyl acetate fraction of Clerodendrum volubile: protocatechuic acid suppresses phagocytic oxidative burst and modulates inflammatory cytokines. Biomed. Pharmacother. 86, 307–315. 10.1016/j.biopha.2016.12.035 28011378

[B14] Hilliard A. Mendonca P. Russell T. D. Soliman K. F. A. (2020). The protective effects of flavonoids in cataract formation through the activation of Nrf2 and the inhibition of MMP-9. Nutrients 12 (12), 3651. 10.3390/nu12123651 33261005 PMC7759919

[B15] Hu Y. Xie S. Wang J. (2022). Advances in the study of cytokines and their signaling pathways associated with posterior cataract. Hans J. Ophthalmol. 11 (2), 193–200. 10.12677/HJO.2022.112027

[B16] Ji B. Yuan K. Li J. Ku B. J. Leung P. S. He W. (2021). Protocatechualdehyde restores endothelial dysfunction in streptozotocin-induced diabetic rats. Ann. Transl. Med. 9 (8), 711. 10.21037/atm-21-1431 33987409 PMC8106075

[B17] Jiang T. Teng S. An X. Yi L. (2022). An overview of the intervention of traditional Chinese medicine in AGEs-RAGE signaling pathway to improve diabetic nephropathy. Glob. Tradit. Chin. Med. 15 (01), 173–178. 10.3969/j.issn.1674-1749.2022.01.042

[B18] Kador P. F. Kinoshita J. H. (1984). Diabetic and galactosaemic cataracts. Ciba Found. Symp. 106, 110–131. 10.1002/9780470720875.ch7 6439498

[B19] Kim Y. S. Kim N. H. Lee S. W. Lee Y. M. Jang D. S. Kim J. S. (2007). Effect of protocatechualdehyde on receptor for advanced glycation end products and TGF-beta1 expression in human lens epithelial cells cultured under diabetic conditions and on lens opacity in streptozotocin-diabetic rats. Eur. J. Pharmacol. 569 (3), 171–179. 10.1016/j.ejphar.2007.05.054 17597607

[B20] Kiziltoprak H. Tekin K. Inanc M. Goker Y. S. (2019). Cataract in diabetes mellitus. World J. Diabetes 10 (3), 140–153. 10.4239/wjd.v10.i3.140 30891150 PMC6422859

[B21] Kopytek M. Ząbczyk M. Mazur P. Undas A. Natorska J. (2020). Accumulation of advanced glycation end products (AGEs) is associated with the severity of aortic stenosis in patients with concomitant type 2 diabetes. Cardiovasc Diabetol. 19 (1), 92. 10.1186/s12933-020-01068-7 32552684 PMC7301463

[B22] Li Y. Huang Z. Pan S. Feng Y. He H. Cheng S. (2023). Resveratrol alleviates diabetic periodontitis-induced alveolar osteocyte ferroptosis possibly via regulation of slc7a11/GPX4. Nutrients 15 (9), 2115. 10.3390/nu15092115 37432277 PMC10181281

[B23] Liu J. Shao H. Gao C. Huo Z. (2023). Research progress on the pathogenesis of macular edema secondary to retinal vein occlusion. Adv. Clin. Med. 13 (4), 5160–5164. 10.12677/ACM.2023.134731

[B24] Liu X. Gong Q. Yang L. Liu M. Niu L. Wang L. (2020). microRNA-199a-5p regulates epithelial-to-mesenchymal transition in diabetic cataract by targeting SP1 gene. Mol. Med. 26 (1), 122. 10.1186/s10020-020-00250-7 33276722 PMC7718685

[B25] Liu Y. W. Liu X. L. Kong L. Zhang M. Y. Chen Y. J. Zhu X. (2019). Neuroprotection of quercetin on central neurons against chronic high glucose through enhancement of Nrf2/ARE/glyoxalase-1 pathway mediated by phosphorylation regulation. Biomed. Pharmacother. 109, 2145–2154. 10.1016/j.biopha.2018.11.066 30551472

[B26] Luo L. Wei Q. Liu L. Lin X. Lin C. Zheng L. I. (2015). Protocatechuic acid benefits proliferation and phenotypic maintenance of rabbit articular chondrocytes: an *in vitro* study. Exp. Ther. Med. 9 (5), 1865–1870. 10.3892/etm.2015.2326 26136906 PMC4471768

[B27] Pang L. Lian X. Liu H. Zhang Y. Li Q. Cai Y. (2020). Understanding diabetic neuropathy: focus on oxidative stress. Oxid. Med. Cell Longev. 2020, 9524635. 10.1155/2020/9524635 32832011 PMC7422494

[B28] Rabbani N. Adaikalakoteswari A. Larkin J. R. Panagiotopoulos S. MacIsaac R. J. Yue D. K. (2022). Analysis of serum advanced glycation endproducts reveals methylglyoxal-derived advanced glycation MG-H1 free adduct is a risk marker in non-diabetic and diabetic chronic kidney disease. Int. J. Mol. Sci. 24 (1), 152. 10.3390/ijms24010152 36613596 PMC9820473

[B29] Rabbani N. Thornalley P. J. (2018). Advanced glycation end products in the pathogenesis of chronic kidney disease. Kidney Int. 93 (4), 803–813. 10.1016/j.kint.2017.11.034 29477239

[B30] Ruiz H. H. Ramasamy R. Schmidt A. M. (2020). Advanced glycation end products: building on the concept of the “common soil” in metabolic disease. Endocrinology 161 (1), bqz006. 10.1210/endocr/bqz006 31638645 PMC7188081

[B31] Semaming Y. Pannengpetch P. Chattipakorn S. C. Chattipakorn N. (2015). Pharmacological properties of protocatechuic Acid and its potential roles as complementary medicine. Evid. Based Complement. Altern. Med. 2015, 593902. 10.1155/2015/593902 PMC4337037 25737736

[B32] Stepp M. A. Menko A. S. (2021). Immune responses to injury and their links to eye disease. Transl. Res. 236, 52–71. 10.1016/j.trsl.2021.05.005 34051364 PMC8380715

[B33] Sun Y. Zhu Y. Liu X. Chai Y. Xu J. (2020). Morroniside attenuates high glucose-induced BMSC dysfunction by regulating the Glo1/AGE/RAGE axis. Cell Prolif. 53 (8), e12866. 10.1111/cpr.12866 32643284 PMC7445400

[B34] Tao D. Liu Z. Wang L. Li C. Zhang R. Ni N. (2022). CircPAG1 interacts with miR-211-5p to promote the E2F3 expression and inhibit the high glucose-induced cell apoptosis and oxidative stress in diabetic cataract. Cell Cycle 21 (7), 708–719. 10.1080/15384101.2021.2018213 35174780 PMC8973334

[B35] Truong C. S. Seo E. Jun H. S. Psoralea corylifolia L. (2019). Psoralea corylifolia L. Seed extract attenuates methylglyoxal-induced insulin resistance by inhibition of advanced glycation end product formation. Oxid. Med. Cell Longev. 2019, 4310319. 10.1155/2019/4310319 31976027 PMC6954480

[B36] Wang Y. H. Han Y. P. Yu H. T. Pu X. P. Du G. H. (2014). Protocatechualdehyde prevents methylglyoxal-induced mitochondrial dysfunction and AGEs-RAGE axis activation in human lens epithelial cells. Eur. J. Pharmacol. 738, 374–383. 10.1016/j.ejphar.2014.04.045 24930813

[B37] Wang Z. Zhang J. Chen L. Li J. Zhang H. Guo X. (2019). Glycine suppresses AGE/RAGE signaling pathway and subsequent oxidative stress by restoring Glo1 function in the aorta of diabetic rats and in HUVECs. Oxid. Med. Cell Longev. 2019, 4628962. 10.1155/2019/4628962 30944692 PMC6421782

[B38] Wei G. Guan Y. Yin Y. Duan J. Zhou D. Zhu Y. (2013). Anti-inflammatory effect of protocatechuic aldehyde on myocardial ischemia/reperfusion injury *in vivo* and *in vitro* . Inflammation 36 (3), 592–602. 10.1007/s10753-012-9581-z 23269534

[B39] Wu H. Chen J. Yang H. (2009). Correlation analysis of salvia miltiorrhiza composition and antioxidant activity. Chin. J. Exp. Traditional Med. Formulae 15 (8), 68–71. 10.3969/j.issn.1005-9903.2009.08.026

[B40] Xing Y. L. Zhou Z. Agula Z. Z. Y. Ma Y. J. Zhao Y. L. Xiao X. H. (2012). Protocatechuic aldehyde inhibits lipopolysaccharide-induced human umbilical vein endothelial cell apoptosis via regulation of caspase-3. Phytother. Res. 26 (9), 1334–1341. 10.1002/ptr.3720 22298410

[B41] Yibo M. (2023). Demethoxycurcumin delay the progression of osteoarthritis via the NRF2-ARE/AGE-RAGE axis. Dalian: Dalian Medical University. 10.26994/d.cnki.gdlyu.2023.000463

[B42] Yun X. (2020). Study on the mechanism of “picking evils in collaterals and treated with phlegm and blood stasis” in Treating pathological changes caused by AGEs-RAGE Axis in DCM rats. Hefei: Anhui University Of Traditional Chinese Medicine. 10.26922/d.cnki.ganzc.2020.000177

[B43] Zhang J. Xiao Y. He J. Song J. Bai Z. Gao S. (2024a). Preventive mechanism of protocatechuic aldehyde on cyclophosphamide-induced acute kidney injury via anti-apoptosis. Drug Eval. Res. 47 (6), 1217–1223. 10.7501/j.issn.1674-6376.2024.06.005

[B44] Zhang Z. Zhao R. Qi Y. (2024b). The role of AGE-RAGE in osteoporosis. Chin. J. Osteoporos. 30 (10), 1483–1486. 10.3969/j.issn.1006-7108.2024.10.014

[B45] Zhu X. Liu H. Liu Y. Chen Y. Liu Y. Yin X. (2020). The antidepressant-like effects of hesperidin in streptozotocin-induced diabetic rats by activating Nrf2/ARE/glyoxalase 1 pathway. Front. Pharmacol. 11, 1325. 10.3389/fphar.2020.01325 32982741 PMC7485173

[B46] Zych M. Wojnar W. Kielanowska M. Folwarczna J. Kaczmarczyk-Sedlak I. (2020). Effect of berberine on glycation, aldose reductase activity, and oxidative stress in the lenses of streptozotocin-induced diabetic rats *in vivo*-A preliminary study. Int. J. Mol. Sci. 21 (12), 4278. 10.3390/ijms21124278 32560082 PMC7349706

